# Latent tuberculosis among pregnant mothers in a resource poor setting in Northern Tanzania: a cross-sectional study

**DOI:** 10.1186/1471-2334-10-52

**Published:** 2010-03-07

**Authors:** Faheem G Sheriff, Karim P Manji, Mohamed P Manji, Mohamedsuhel M Chagani, Rose M Mpembeni, Ahmed M Jusabani, Zaheerabbas R Alwani, Taha S Karimjee

**Affiliations:** 1 School of Medicine, Muhimbili University of Health and Allied Sciences, Dar es Salaam, Tanzania; 2 Paediatrics and Child Health, School of Medicine, Muhimbili University of Health and Allied Sciences, Dar es Salaam, Tanzania; 3 Biostatistics and Epidemiology, School of Public Health, Muhimbili University of Health and Allied Sciences, Dar es Salaam, Tanzania; 4 Diagnostic Radiology, Tumaini University - KCMC, Moshi, Tanzania; 5 School of Medicine, Ryazan State I P Pavlov Medical University, Ryazan, Russia; 6 School of Medicine, Nizhny Novgorod State Medical Academy, Nizhny Novgorod, Russia

## Abstract

**Background:**

Untreated latent TB infection (LTBI) is a significant risk factor for active pulmonary tuberculosis, hence predisposing to adverse pregnancy outcomes and mother to child transmission. The prevalence of latent tuberculosis in pregnancy and its association, if any, with various socio-demographic, obstetric and clinical characteristics was evaluated.

**Methods:**

Northern Tanzania was chosen as the study site. In a cross-sectional study, a total of 286 pregnant women from 12 weeks gestational age to term were assessed. Screening was undertaken using an algorithm involving tuberculin skin testing, symptom screening in the form of a questionnaire, sputum testing for acid fast bacilli followed by shielded chest X-rays if indicated. HIV serology was also performed on consenting participants.

**Results:**

Prevalence of latent infection ranged between 26.2% and 37.4% while HIV sero prevalence was 4.5%. After multivariate logistic analysis it was found that age, parity, body mass index, gestational age, and HIV sero status did not have any significant association with tuberculin skin test results. However certain ethnic groups were found to be less vulnerable to LTBI as compared to others (Chi square = 10.55, p = 0.03). All sputum smears for acid fast bacilli were negative.

**Conclusion:**

The prevalence of latent tuberculosis in pregnant women was found to be relatively high compared to that of the general population. In endemic areas, socio-demographic parameters alone are rarely adequate in identifying women susceptible to TB infection; therefore targeted screening should be conducted for all pregnant women at high risk for activation (especially HIV positive women). As opposed to the current policy of passive case detection, there appears to be an imminent need to move towards active screening. Ethnicity may provide important clues into genetic and cultural differences which predispose to latent tuberculosis, and is worth exploring further.

## Background

It is estimated that TB infection is present in one-third of the world's population, or 2 billion people. Eight million new cases of active disease have been estimated to occur worldwide annually[1]. The prevalence of LTBI in Sub-Saharan Africa is 31%[2], while that of HIV is 5%-35% of the adult population; one-third to one-half of HIV-infected individuals are co-infected with *Mycobacterium tuberculosis*[1].

The incidence of tuberculosis among women in South Africa (mean age 33.4 years) increased from 154/100,000 to 413/100,000 between 1991 and 1995; 44% of this increase was attributable to HIV-1 infection [3]. Similar increases in notification in women were reported in Tanzania between 1985 and 1991 [4] in keeping with the global increase in tuberculosis.

In a South African study, TB was the third leading cause of maternal mortality, mostly in combination with HIV-infection[5]. Neonatal mortality and extreme prematurity have also increased significantly due to the problem[6]. Tanzania has not been spared.

Women between the ages of 15 and 49 years carry the greatest risk of converting from tuberculosis infection to disease[7]. It is expected that the incidence of latent tuberculosis among pregnant women would be as high as in general population, although no studies have been conducted to substantiate this fact. The rate of active TB in pregnant women ranges from 0.1% to 1.9% [8]. Furthermore, the risk of mother-to-child-transmission for active disease is 15% within 3 weeks of delivery [9].

The usefulness of the Tuberculin Skin Test (TST) in diagnosing LTBI has always been debated, especially since the introduction of more sensitive interferon gamma (IFN gamma) assays. While research has shown that pregnancy does not alter tuberculin reactivity and that this test is entirely safe and reliable for pregnant women [10], the same has not been substantiated for IFN-gamma assays. Skin testing is also cheaper and more readily available in resource poor settings.

Despite the amount of research conducted on screening for active TB there has been scanty work done on latent tuberculosis among pregnant women in developing countries. Hence the need for a study aimed at evaluating the prevalence of LTBI in pregnancy and the association between various socio-demographic/obstetric parameters and latent TB infection is evident. Chest radiography was included as part of the work up of TST positive pregnant women, especially since it is vital to rule out active TB before initiation of Isoniazid Prophylactic Therapy (IPT).

## Methods

### Study Area

An urban hospital based cross-sectional study was undertaken in Arusha city, the capital of Arusha region. This region was selected since its TB notification rates are presumed to be representative of the whole nation. Arusha region accounted for about 7% of the total reported TB cases in the year 2004[11]. It has therefore been an important focus in the nationwide campaign against tuberculosis by the National TB and Leprosy Program (NTLP).

The "Maasai" are the most influential tribe in the region. With urbanization, Arusha is attracting people of many other tribes including the "Chagga", "Sambaa", "Nyaturu" and "Pare". Most speak Kiswahili and their tribal languages. Ngarenaro ward, which was the actual study site, is a cosmopolitan area situated on the outskirts of the city and ward estimates of 2002 reflect a total population exceeding 15,700.

### Study Population

The study population included pregnant women attending the Ngarenaro antenatal care clinic (ANC) between June and August 2008. This is the largest of its kind in Arusha region. It records an annual attendance of between eight and ten thousand pregnant women.

### Screening Methods

Consenting pregnant women were first subjected to a TB symptom check-list (administered in Swahili by bi-lingual researchers) followed by a routine physical examination. They were also subjected to the Mantoux test which involved injection of 0.1 ml(5 TU) of PPD RT 23 SSI intradermally on the volar surface of the fore-arm, midway between the elbow and wrist joints. A 27 G syringe was used and a wheal between 5 and 10 mm was produced. The injection site was marked with a waterproof pen and the date and time noted. Research participants were advised neither to scratch nor to apply any soap or chemical solutions to the site. Results were interpreted 48 hours later using the ball-point pen method described by Sokal[12]. At the initial visit, those with symptoms/signs consistent with suspected tuberculosis had then their sputum screened for Acid Fast Bacilli (AFB) by a spot sample followed by a morning sputum sample collected in accordance with revised NTLP guidelines. Specimens were processed by a centrifugation technique known to improve AFB yield (sensitivity)[13]. The slides were interpreted with the assistance of a senior laboratory technician at the Kilimanjaro Christian Medical Centre (KCMC) which is the nearest tertiary referral hospital. Next, HIV serology was carried out on consenting participants using a combination of SD-Bioline^® ^and Determine^® ^rapid antibody test kits according to the National Guidelines for prevention of mother-to-child transmission. Finally, participants who presented with a productive cough/prolonged cough(>2 weeks), found to be either HIV positive or TST positive were subjected to a single postero-anterior Chest Radiograph (CXR) with abdominal shielding. The CXRs were interpreted by an experienced radiologist working at the regional hospital, and re-interpreted by a consultant radiologist at the KCMC referral hospital. The latter was blinded to the findings of the former. The Helsinki declaration was abided by throughout the research process.

### Sample Size

The following formula was used for calculation of sample size:

Where N = minimum sample size

Z = 1.96 (95% confidence interval)

P = prevalence (31% LTBI in Africa) [2]

d = Margin of error = 0.05

Allowing for a 20% failure to follow up, a minimum sample size of 395 was reached.

### Statistical Analysis

Three (3) methods were used to determine the prevalence of Latent TB infection (LTBI). The first method factored in the proportion of all indurations > = 14 mm multiplied by 1.22 to make up for the reduced sensitivity associated with cut off points higher than those recommended by the United States Center for Disease Control (US CDC) [14]. The second method counted indurations at the mode(17 mm) once and all indurations above the mode(> = 18 mm) counted twice as an estimate of the number of infections. This method is known as the mirror image (MI) method [14]. The third method which is advocated by the US Centre for Disease Control made use of predefined cut off points (> = 10 mm induration for HIV negative participants in endemic areas for TB; > = 5 mm for HIV positive participants or those with evidence of healed TB lesions on CXR) [15]. This latter definition of LTBI was utilized in the analysis of socio-demographic/obstetric characteristics associated with TB infection in order to achieve uniformity when comparing with previous studies. In all three methods utilized, patients with radiologic features suggestive of active TB were excluded.

The data was initially processed using the Epi-Info v.6 statistical package. It was then re-analyzed using SPSS v. 13.0 to enable multivariate logistic regressions to be carried out. Pearson's Chi-squares, p-values(including Fischer's exact values when appropriate) and Odd's Ratios were calculated. 2-tailed p-values of less than 0.05 were considered significant. Flow diagrams, tables and bar charts were constructed to summarize key data.

### Inclusion criteria

All consenting pregnant women with gestational ages 12 weeks till term were included, on an "intention to treat" basis.

### Ethical considerations

Ethical clearance was obtained from the MUHAS Community Health Department. Verbal informed consent, witnessed by a registered nurse, was obtained from each participant prior to enrollment into the study. All participants with chest radiographic features suggestive of active TB and HIV positive participants with LTBI were referred to the District TB and Leprosy Coordinator (DTLC) for further evaluation and treatment in accordance with NTLP guidelines.

## Results

### Socio demographic profile

Of the 407 women approached to enroll in the study, 396 consented and 11 refused to give consent for various reasons. 286 (72.2%) returned for Tuberculin Skin Tests (TSTs) readings. Subjects who were lost to follow up were not socio-demographically different from those who returned in relation to parity and gestational age(p = 0.63 and p = 0.35 respectively). However, non respondents were younger (mean 23.71 yrs) compared to respondents (mean 25.33 yrs; p = 0.04).

The median age of those screened was 25 years, ranging from 16 to 40 years. The mean Body Mass Index (BMI) for the study population was 25.03 kg/m^2^. (95% CI = 24.49 - 25.56 kg/m^2^). The gestational age of participants ranged from 12 to 40 weeks with a mean of 26.2 weeks (95% CI = 25.4, 26.9); while parity ranged from 0 (nulliparity) to 6 with a median of 1.

### Prevalence of LTBI and HIV

**Figure 1:**
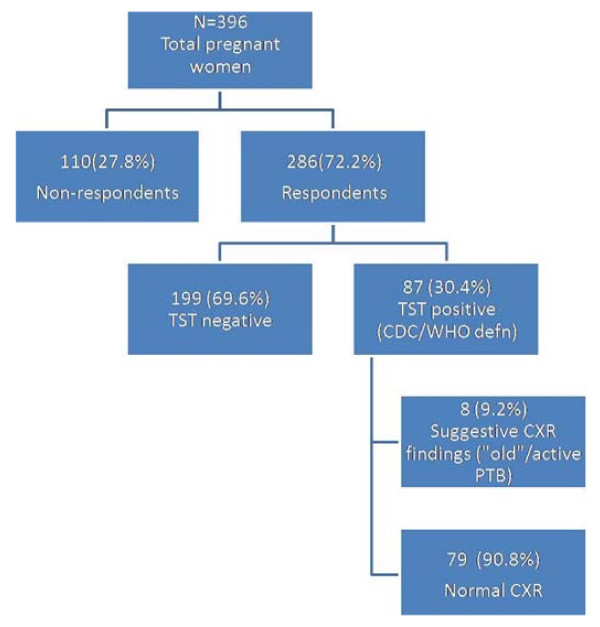
**Flow diagram representing all pregnant women screened**.

**Figure 2:**
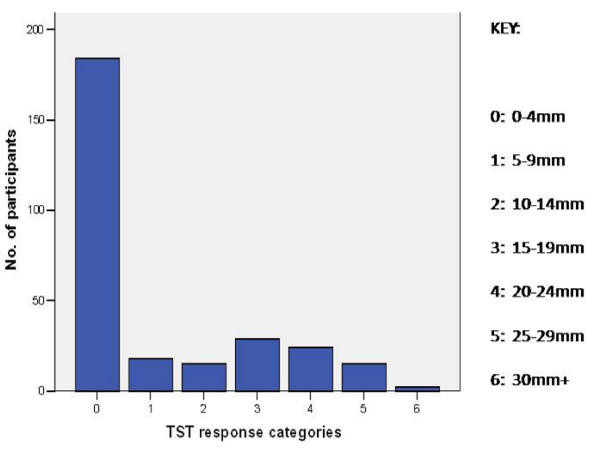
**Bar chart showing TST induration sizes and corresponding frequencies**.

Using the first method (cutoff at 14 mm) the prevalence of latent TB infection was 26.2%(75 of 286). Using the second method (mirror image with a mode of 17 mm), the prevalence of infection stood at 37.4%. Using the third method(US CDC cutoff points), the prevalence of TST positive pregnant women was 30.4%(87 of 286) while that of latent TB infection was 29.7%(85 of 286). Two women, both HIV negative, were excluded from the latter group since they were found to have CXR findings highly suggestive of active TB. The mean TST induration measured 6.36 mm (95% CI 5.27 - 7.45 mm). Table 1, Fig. 1 and Fig. 2 below summarize the findings.

**Table 1:** Prevalence of LTBI and HIV infection among pregnant women attending ante-natal care clinics (N = 286)

LATENT TB INFECTION (US CDC)	Frequency	Percent
Present	85	29.7%

Absent	201	70.3%

**HIV SERO STATUS**	**Frequency**	**Percent**

Positive	13	4.5%

Negative	273	95.5%

NB: Latent TB infection is not synonymous to a positive TST reading, since all those with chest radiographic features suggestive of active TB were excluded from the LTBI category.

The prevalence of HIV infection in the study population was 4.5%. No significant association was noted between HIV sero status and the size of TST response (p = 0.93; TST responses graded at 5 mm intervals).

One of the HIV infected women was diagnosed to have TB lymphadenitis using fine needle aspiration cytology of the axillary lymph nodes. She had a negative TST but a highly suggestive chest radiograph for pulmonary TB.

### TST results versus socio demographic parameters

On univariate analysis, no significant correlation was observed between LTBI (latent TB infection) and age>30(p = 0.15), marital status(p = 0.14), parity(p = 0.63) or gestational age(p = 0.49). Certain ethnic tribes were found to have a lower prevalence of LTBI than others-these included the "Sambaa"(OR = 0.21, 95% CI = 0.05, 0.97; p = 0.045) and "Chagga"(OR = 0.47, 95% CI = 0.25, 0.92; p = 0.03). Ethnicity as a vulnerability factor retained significance on multivariate analysis(Chi-square 10.55, p = 0.03). Table 2 below highlights these results.

**Table 2:** TST results versus various socio demographic factors [multivariate logistic regression] (N = 286)

INPUT VARIABLES	OUTCOMES	TST POSITIVE	TST NEGATIVE	p-VALUE	ODDS RATIOS	95% CI Lower Bound	95% CI Upper Bound
AGE	> = 30 yrs	23(35.4%)	42(64.6%)	.147	1.573	.853	2.899
	
	<30 yrs	63(28.4%)	159(71.6%)	.	.	.	.

HIV STATUS	HIV NEGATIVE	84(30.8%)	189(69.2%)	.460	1.658	.443	6.345
	
	HIV POSITIVE	3(23.1%)	10(76.9%)	.	.	.	.

ETHNIC TRIBE	CHAGGA	16(21.6%)	58(78.4%)	**.027**	**.474**	**.247**	**.919**
	
	PARE	8(29.6%)	19(70.4%)	.664	.819	.332	2.019
	
	NYATURU	11(39.3%)	17(60.7%)	.640	1.222	.528	2.830
	
	SAMBAA	2(10.5%)	17(89.5%)	**.045**	**.214**	**.047**	**0.968**
	
	OTHERS	50(36.2%)	88(63.8%)	.	.	.	.

NB

a The last outcome from each category was used as the reference category during multinomial regression

b Row percentages add up to 100%

### TST results versus chest radiographic findings

An overall 9.2%(8 of the 87) TST positive individuals yielded CXR abnormalities suggestive of TB (healed or active). Fig. 1 below represents these findings. Radiologic features suggestive of active PTB were detected in 1.4% of the total population screened.

All 28 participants whose sputum was screened had AFB negative smears on two consecutive occasions, despite the use of a centrifugation technique during specimen processing.

## Discussion

### Prevalence of LTBI and HIV among pregnant women

The prevalence of LTBI among pregnant women using the three methods was 26.2%, 37.4% and 29.7% respectively. While two of these estimates do not differ much from that of the general population in Sub Saharan Africa (31%)[2]; the mirror image estimate (37.4%) is considerably higher. Since the positive predictive value (PPV) of any diagnostic test increases with increasing disease prevalence, it is worthwhile noting that skin testing is more useful as a screening test for TB in endemic regions such as this study site. Most cases of latent TB infection (LTBI) convert to active TB within the first 2 years of infection [15]. Acquisition of TB infection is highest during the reproductive age group and pregnancy does not alter the immunological reactivity to tuberculin[16]. Considering the relatively high latent TB prevalence, it could reasonably be speculated that active TB in this subpopulation would also be high. The estimate arrived at in this study (1.4%) is closer to the upper limit of the range suggested by previous research conducted among pregnant women (0.1%-1.9%) [8]. Since active PTB was diagnosed by radiology and microbiologic confirmation with cultures not undertaken due to logistic constraints, this estimate should be regarded with caution. In any case it helps explain the observed high rate of LTBI owing to the fact that the aforementioned entities i.e. latent and active TB are intimately related in a vicious cycle. These findings should therefore raise sufficient concern to prompt further research followed by appropriate intervention due to the fact that TB is becoming an increasingly important cause of non-obstetric mortality and adverse pregnancy outcomes [17]. Furthermore, the risk of mother-to-child-transmission for active disease is 15% within 3 weeks of delivery [9]. This makes it an emerging problem of public health significance.

No significant difference was found between HIV positive and negative subjects as far as the size of TST response was concerned (p = 0.93). So, although HIV positive status may cause a failure to mount an immune response due to cutaneous anergy, it does not diminish the size of the response[18,19]. Recent studies do suggest that it is mainly the TST positive HIV infected group (high TB risk) that stands to benefit most from treatment of LTBI as compared to the non-converters[20,21]. This explains the stance of the WHO which recommends treatment of LTBI in HIV positive individuals only if found to be TST positive[22].

### LTBI in relation to socio demographic characteristics

Previous prevalence studies for LTBI have reported a positive trend of TST results with age reflecting cumulative exposure to *M. tuberculosis *with time [23]. In contrast, this study did not show a significant difference in the prevalence of LTBI in the <30 age group compared to the > = 30 age group(p = 0.15). The same observation has been reported by a number of South African studies and probably reflects a high incidence of TB in the young population due to hyper endemicity [24,25]. The lack of significant association between various socio demographic/obstetric characteristics (age, marital status, area of residence, parity, gestational age, BMI) and TST results implies that in endemic areas for TB there are no obvious features to identify women at higher risk for acquisition of LTBI. In a South African study, no significant differences were found between those who were diagnosed with tuberculosis versus those who were not as far as age and parity were concerned (p = 0.9 and 0.8 respectively)[5]. Due to these challenges, screening should target pregnant women at risk for activation (rather than acquisition) of latent infection. Women at risk for activation include but are not limited to immunocompromized individuals, the most common subpopulation being HIV positive women. Although not as common among pregnant women; malnutrition, diabetes mellitus, silicosis, steroid therapy and chronic renal failure are also risk factors[8].

The only socio-demographic parameter which was strongly associated with LTBI was ethnicity. Certain tribes appeared to be less vulnerable to acquiring latent TB infection; these included the "Chagga" and the "Sambaa". These findings retained significance after multivariate analysis and even after area of residence was factored in. A possible explanation may revolve around the better socio-economic status of the Chagga due to the fertile slopes of Mount Kilimanjaro and Meru as well as their age-old knowledge on irrigation and good farming practices[26] resulting in an improved standard of living with a corresponding decrease in the household crowding index. The other major groups participating in the study were the "Maasai", "Pare" and "Nyaturu" tribes and did not appear to enjoy the same protective benefit. The Maasai and other tribes around Arusha region (Iraqws and Barbaigs) have a practice of drinking raw bovine milk and blood which often exposes them to infection with *Mycobacterium bovis *[27]; this may then produce a positive TST reading confounding infection with *Mycobacterium tuberculosis*. The importance of ethnicity in interpretation of TST results has been highlighted by a couple of recent studies[28,29]. One such recent study in Laos, Asia revealed that children living in ethnic minorities were at significantly higher risk of acquiring LTBI compared to the rest [29]. Our study is among the first to report related findings among adult females of reproductive age (pregnant women).

### Tuberculin skin testing and chest radiography

An abnormal chest radiograph is highly sensitive for tuberculosis in immunocompetent individuals as demonstrated by Good JT [30]. In this study only 9.2% of all TST positive participants had suggestive radiologic abnormalities, of which only two showed features of active TB. These findings may be comparable to a study in India which was investigating the efficacy of chest radiography in screening for active TB among HIV positive pregnant women [31]. It found that that of those pregnant women with either a positive symptom screen or a positive TST, only 9.0% had features suggestive of active TB on chest radiography. They concluded that the latter did not improve sensitivity nor specificity in the screening process and was therefore unnecessary in the absence of symptoms[31].

### Strengths and limitations of the study

The study was conducted among pregnant women attending ante-natal care clinics, and therefore could be conveniently followed up. In addition the study techniques were cost effective while at the same time meeting international standards for research methodology. These factors may facilitate reproducibility in resource-limited settings.

Study limitations included a higher-than-expected loss to follow up despite adequate compensation. This may have introduced an element of selection bias. However as demonstrated by this study and a couple of others, age is not associated with the prevalence of LTBI (latent TB infection) in high incidence areas[24,25]. Since respondents and non-respondents only differed as far as age was concerned all other variables being similar, its effect on the validity of this study would be minimal at best. Finally, since only pregnant women were enrolled, the study results may not be generalizable to the whole population.

## Conclusion

The prevalence of latent TB infection among pregnant women in Northern Tanzania was found to be relatively high (26.2%-37.4%). This is reason for concern since recent acquisition of latent infection is among the most important risk factors for active disease, and hence for pregnancy related complications, mother to child transmission and subsequently neonatal tuberculosis. These findings necessitate serious thought on the issue of active screening for LTBI and subsequent Isoniazid Prophylactic Therapy (IPT), as opposed to passive case detection. With sufficient funding and proper implementation, this change in policy has the potential to significantly enhance TB control in Tanzania and possibly Sub Saharan Africa. Since in endemic areas socio-demographic characteristics alone rarely help identify women predisposed to TB infection, targeted screening should be conducted for all pregnant women at risk of activation (in particular HIV positive women). Ethnicity is a lead worth exploring in future research since underlying issues related to genetics, socio-economic differences and high risk cultural practices will be brought to surface and therefore hopefully addressed.

## Abbreviations

AFB: Acid-fast bacilli; ANC: Ante-natal care clinic; CXR: Chest radiograph; DTLC: District TB and Leprosy Coordinator; IFN-gamma: Interferon gamma assay; LTBI: Latent TB infection; NTLP: National TB and Leprosy Program; PPD: Purified Protein Derivative; TB: Active tuberculosis; TST: Tuberculin Skin Test; TU: Tuberculin Unit; US CDC: United States Center for Disease Control.

## Competing interests

The authors declare that they have no competing interests.

## Authors' contributions

FGS and KPM were involved in designing the methodology, data analysis and discussion of the paper. MPM and MMC assisted in literature review and data analysis. RNM offered expert advice and help as far as biostatistics and epidemiology was concerned. AMJ assisted with radiologic interpretation of chest X-rays. FGS, ZRA and TSK were involved in the planning and technical aspects of research and data collection.

## Pre-publication history

The pre-publication history for this paper can be accessed here:

http://www.biomedcentral.com/1471-2334/10/52/prepub
